# Subungual Glomus Tumor With Normal Findings on Imaging: A Case Report

**DOI:** 10.7759/cureus.102017

**Published:** 2026-01-21

**Authors:** Youssef A Abdelmegeid, Ahmed Yousry Saber, Victoria Deans

**Affiliations:** 1 Medicine and Surgery, Manchester University NHS Foundation Trust, Manchester, GBR; 2 Trauma and Orthopedics, Gateshead Health NHS Foundation Trust, Exeter, GBR; 3 Trauma and Orthopaedics, Calderdale and Huddersfield NHS Foundation Trust, Huddersfield, GBR

**Keywords:** diagnosis, glomus tumor, mri, subungual, surgical excision

## Abstract

Glomus tumors are frequently associated with cold sensitivity, pain, and tenderness. They are benign tumors arising from the glomus body and typically present with subungual pinpoint pain. Diagnosis is primarily based on clinical findings, with ultrasound or MRI often used to confirm the diagnosis. We report the diagnosis and surgical management of a 49-year-old female with a classic glomus tumor. Despite normal ultrasound and MRI findings, she was diagnosed based on history and physical examination. Surgical removal of the lesion resulted in complete resolution of her symptoms, and histology subsequently confirmed the diagnosis of a glomus tumor. Clinicians should suspect a glomus tumor based on clinical findings, and a normal ultrasound or MRI should not exclude its presence.

## Introduction

Glomus tumors originate from the neuromyoarterial cells of the glomus body in the reticular dermis and are typically benign [1]. Most occur in the upper extremities, accounting for about 2% of all hand tumors [2]. Approximately 50% arise in the subungual region, where the proliferation of cells in this confined space typically causes severe pain. Histologically, glomus tumors consist of glomus bodies and are usually less than one centimeter in size. Glomus bodies regulate blood flow and temperature, and subungual tumors often present as a blue papule accompanied by hyperalgesia to pressure and cold [3]. Surgical removal of the lesion results in complete symptom resolution [4]. Although ultrasound and MRI have a sensitivity of approximately 90%, there is a high reliance on imaging as a useful diagnostic tool [5]. Here, we report a clinically typical case of a glomus tumor with normal ultrasound and MRI findings.

## Case presentation

A 49-year-old female presented to orthopedics with a two-year history of discomfort and marked tenderness over the nail bed of her left middle finger, particularly when exposed to cold or subjected to slight pressure. The pain was constant and severely debilitating, interfering with daily activities; she reported difficulty using her hand and was unable to hold her daughter’s hand. She had not noticed any deformity or nail changes and was otherwise in good health. Her past medical history included mild carpal tunnel syndrome, which had resolved with the use of night splints. She works as a warehouse packer, requiring frequent repetitive hand movements, which she could manage slowly despite the pain.

On examination, the nail plate appeared normal, but there was marked tenderness at the base of the nail plate in the lunular region on the ulnar side. The distal interphalangeal joint had a normal range of motion. Neurological examination revealed mildly reduced light-touch sensation over the ulnar border of the middle finger. Nerve conduction studies suggested mild carpal tunnel syndrome, but this did not fully explain her symptoms.

**Figure 1:**
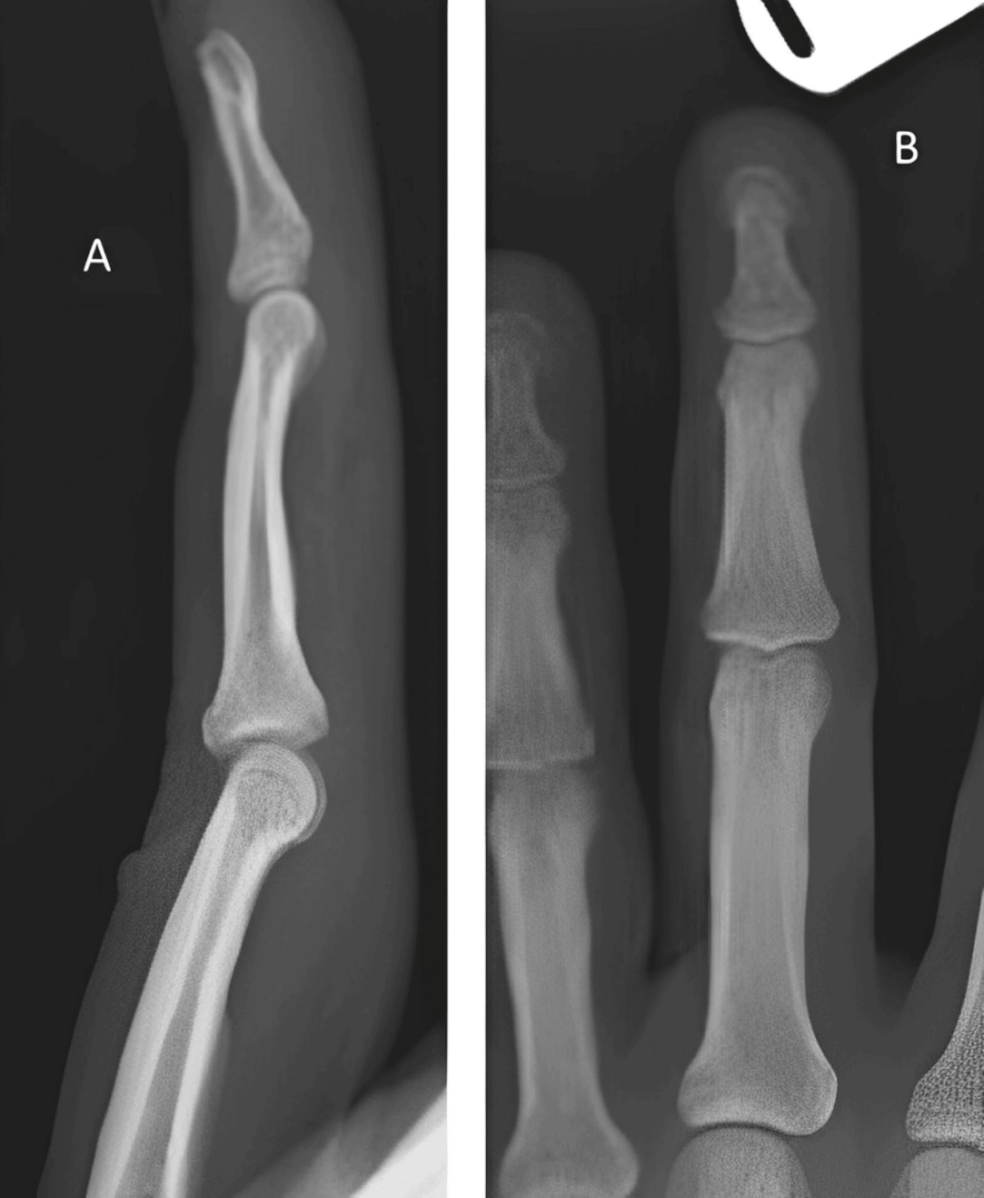
Normal radiograph of the middle finger (A) Sagittal view. (B) Posteroanterior view.

**Figure 2:**
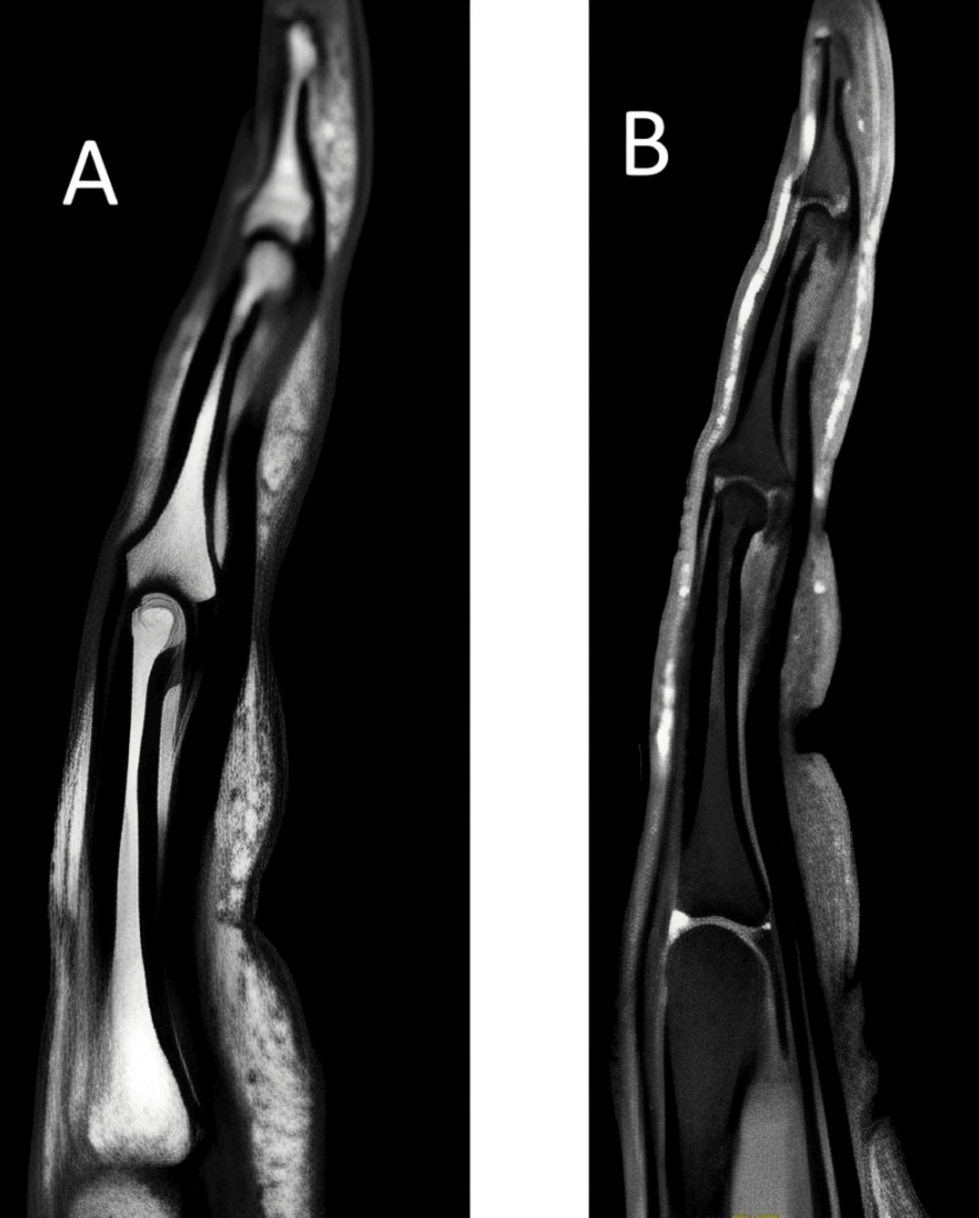
Normal MRI of the middle finger (A) T1-weighted image showing no lesion. (B) T2-weighted image showing no lesion.

Radiographs of the finger were normal (Figure 1), as was the ultrasound. An MRI of the left hand was also reported as normal (Figure 2). The MRI included sagittal and axial series through the index, middle, and little fingers, with additional post-contrast images. No abnormalities were identified, and specifically, there was no evidence of a glomus tumor or neuroma.

This case highlights the discrepancy between normal imaging and clinical findings, emphasizing that a glomus tumor may still be present despite unremarkable radiological studies.

At the next clinic follow-up, the patient had developed ridging of the nail plate, a sign of a compressive lesion at the germinal matrix, as such damage can result in this ridging. She described her symptoms as severe and requested fingertip amputation. In a frank discussion, we explained that, on rare occasions, a glomus tumor may be too small to detect on MRI or ultrasound and that exploratory surgery could identify a tumor. We also clarified that we could not guarantee that surgical excision would fully alleviate her symptoms.

It was agreed to proceed under a local anesthetic ring block to remove the nail plate, excise the area of nail bed corresponding to the well-localized tenderness, repair the nail bed, and temporarily reapply the nail plate to maintain lunular patency. The excised tissue would be sent for histology. Fingertip amputation was reserved as a secondary, last-resort option. Surgical risks, including infection, persistent nail plate ridging, bleeding, and recurrence, were discussed, and the patient consented.

In December 2019, she underwent excision of the lesion on the left middle finger under an 8 mL ring block using 0.5% Chirocaine. Two oblique proximal incisions were made from the base of the nail plate, and the nail plate was removed. A small area of darker, discolored nail bed was observed beneath the eponychium, adjacent to the germinal matrix, corresponding to the area of pain and tenderness.

The lesion was excised down to the distal phalanx and sent for histology. The longitudinal nail bed defect was then repaired, the wound was irrigated, and the nail plate was temporarily reapplied. Inadine dressing and finger protection were applied.

Histology revealed a well-circumscribed subepithelial lesion composed of capillary-sized blood vessels surrounded by a monotonous population of cells with round, dark nuclei arranged in nests within a loose fibrous stroma. No atypical features were observed, and the overlying squamous epithelium appeared normal. The findings were consistent with a glomus tumor, which appeared completely excised, with no evidence of dysplasia or malignancy.

At the two-week follow-up, she reported significant pain reduction. There was no pain at rest, though occasional dull pain occurred with pressure. The wound was clean and dry, and the nail plate remained in situ. She was advised to monitor carefully for signs of infection, as excision had extended down to the distal phalanx, making infection a serious concern.

At eight weeks postoperatively, she was entirely pain-free and much happier. She had experienced a superficial infection, successfully treated with oral antibiotics. The new nail plate had begun to grow through, and she was discharged with open access for follow-up. The histology confirmed a glomus tumor, and she was reminded of the small risk of recurrence. Overall, she was very satisfied with the outcome.

## Discussion

The glomus body is the thermoregulatory apparatus of the cutaneous microvasculature and functions as an arteriovenous anastomosis. A hamartomatous lesion of this apparatus results in a benign tumor, known as a glomus tumor [6]. It represents a hyperplasia of the glomus body. Masson first described the pathological findings in 1924, characterizing them as tumors arising from the neuro-myo-arterial body [2]. Other names for glomus tumors include glomangioma, tumor of the neuro-myo-arterial glomus, and angio-neuro-myoma [7]. Glomus tumors most commonly occur beneath the nails, along the sides of the fingers, and on the palms [7].

Shifts in temperature have been suggested to cause the myofilaments within the lesion to contract, thereby increasing the pressure inside the capsule and generating pain. The mechanism of pain in glomus tumors is not fully understood and remains an area for further research [8]. Several mechanisms have been proposed to explain glomus tumor-associated pain. First, some researchers suggest that the pain arises from the abundant unmyelinated sensory nerve fibers within the lesion [9]. Second, the tumor’s capsule may make it particularly sensitive to pressure. Third, the large number of mast cells within the tumor can release mediators that heighten the sensitivity of nearby mechanoreceptors and thermoreceptors. These factors can elicit severe pain even in response to ordinary stimuli [10]. An identified contributor to pain is the predominance of non-myelinated nerve fibers penetrating the tumor [10]. Clinically, glomus tumors are characterized by cold hyperalgesia, severe intermittent pain, and localized tenderness [6].

Several tests are used to diagnose a glomus tumor. Love reported that localization of tenderness to a pinhead-sized area is suggestive of a glomus tumor [11]. Love’s pin test is considered positive when the patient experiences intense pain after the area over the tumor is pressed with a ballpoint pen, pinhead, or K-wire. Hildreth's test is also reported to be a reliable clinical sign for diagnosis [12]. It is performed by elevating the patient’s arm to exsanguinate it and inflating a tourniquet to 250 mm Hg [13]. The test is positive if this results in a reduction of pain and tenderness or if there is a sudden onset of pain in the tumor area when the cuff is released [7].

For imaging diagnosis, ultrasonography may be preferable to MRI in terms of time, cost, and the ability to evaluate lesions dynamically [10]. Radiographs can show cortical thinning and erosive changes in the adjacent bone [1]. On MRI, glomus tumors appear as low-intensity lesions on T1-weighted images, with enhancement after gadolinium injection, and as hyperintense on T2-weighted images [1]. MRI has a sensitivity of 90% for glomus tumors but only 50% specificity [2], though it remains helpful for tumor localization [4]. Multidetector CT can directly demonstrate a subungual nodule and depression of the distal phalanx, aiding in tumor localization [2]. This case describes a patient with a histologically confirmed glomus tumor who had normal ultrasound and MRI scans. Differential diagnoses include subungual exostosis, neuroma, ganglion, inclusion cyst, and melanoma [8].

Subungual melanoma typically presents as a dark longitudinal streak extending from the base of the nail bed to the tip of the nail plate [14]. Treatment usually involves excisional biopsy or, occasionally, local amputation with or without adjuvant therapy [15]. Early recognition is critical, as delayed treatment worsens prognosis. Any pigmented lesion that does not advance with nail growth or persists beyond six weeks warrants investigation.

Glomus tumors are immunoreactive for smooth muscle actin and CD34 [16]. Histological examination of the tumor shows variable amounts of vascular tissue, smooth muscle, and glomus cells. Depending on the predominant component, these lesions are classified as glomangiomas, solid glomus tumors, or glomangiomyomas [1]. Complete intraoperative excision is essential to reduce recurrence rates [3]. Thorough surgical removal is critical to minimize recurrence, and bone curettage is performed if the tumor extends into the bone. Nail removal is carried out for centrally located tumors, while en bloc resection is indicated if malignancy is suspected [10]. Microscope-assisted surgery, though technically more demanding, offers advantages such as minimizing tissue damage and improving the likelihood of complete tumor excision [2]. Long-term follow-up over 12 years has shown that this approach is as safe as traditional surgery and has a low recurrence rate due to more complete excision [3]. The reported incidence of tumor recurrence after surgical excision varies widely, ranging from 5% to 50%, primarily depending on the surgical technique employed [7]. In addition, intraoperative assessment for multiple tumors is important.

## Conclusions

This case emphasizes that clinical suspicion and careful physical examination are essential for diagnosing glomus tumors. Although imaging can be a valuable tool, it may provide false reassurance and should never outweigh strong clinical findings and physician judgment. Delayed or incorrect diagnosis can result in unnecessary patient suffering. Despite being a single case, this report underscores the importance of considering a broad range of differential diagnoses. We describe a patient with normal ultrasound and MRI findings whose glomus tumor was ultimately confirmed histologically.
